# The effect of aging on pacing strategies of cross-country skiers and the role of performance level

**DOI:** 10.1186/s11556-018-0193-y

**Published:** 2018-04-25

**Authors:** Pantelis Theodoros Nikolaidis, Elias Villiger, Thomas Rosemann, Beat Knechtle

**Affiliations:** 1 Exercise Physiology Laboratory, Nikaia, Greece; 2 Institute of Primary Care, University of Zurich, Zurich, Switzerland; 3 Medbase St. Gallen Am Vadianplatz, Vadianstrasse 26, 9001 St. Gallen, Switzerland

**Keywords:** Age, Endurance exercise, Gerontology, Sport performance, Winter sport

## Abstract

**Background:**

The participation of master cross-country (XC) skiers in training and competition has increased during the last decades; however, little is known yet about whether these athletes differ from their younger counterparts in aspects of performance such as pacing. Therefore, the aim of the present study was to examine the combined effect of age and performance (race time) on pacing in cross-country (XC) skiing. We analyzed all finishers (*n* = 79,722) in ‘Vasaloppet’ from 2012 to 2017, the largest cross-country skiing race in the world, classified according to their race time into 10 groups: 3-4 h, 4-5 h, ..., 12-13 h.

**Results:**

A trivial main effect of sex on total pace range was observed (*p* < 0.001, η^2^ = 0.002), where women (44.1 ± 10.2%) had larger total pace range than men (40.9 ± 11.8%). A large main effect of performance group on total pace range was shown (*p* < 0.001, η^2^ = 0.160), where the smallest total pace range was 21.8 ± 1.9% (3-4 h group) and the largest 50.1 ± 9.9% (10-11 h group). A trivial sex×performance group interaction on total pace range was found (*p* < 0.001, η^2^ = 0.001) with the largest sex difference in pacing shown in 9-10 h group. A trivial and small main effect of age was found in women (*p* < 0.001, η^2^ = 0.005) and men (*p* < 0.001, η^2^ = 0.011), respectively, where the masters had smaller total pace range than their younger counterparts. A trivial age group×performance group interaction on total pace range was observed in both women (*p* < 0.001, η^2^ = 0.008) and men (*p* < 0.001, η^2^ = 0.006) with smaller differences among age groups in the faster performance groups.

**Conclusions:**

In summary, master XC skiers adopted a relatively even pacing independently from their race time and the differences in pacing from the younger XC skiers were more pronounced in the slower masters. These findings suggest that exercise attenuates the decline of performance in master XC skiers as shown by the similar pacing strategies between fast master XC skiers and their younger counterparts.

## Background

Cross-country (XC) skiing is one of the most popular winter endurance sports, especially in countries in the central and north Europe. Considering the increased participation of master XC skiers in training and competition, an increased scientific interest has been focused on performance aspects such as age of peak performance and variation of sex difference by age [1–4]. The average age of women and men XC skiers in a very popular race, Engadin ski marathon, is 38 and 44 years old, respectively [3]. Men are faster than women by ~ 16% [3] and the age of peak performance in both sexes is ~ 40 years [2]. After this age, performance decreases steadily in both sexes [1, 4].

Another aspect of performance is pacing [5]; however, limited research on pacing in XC skiing has been conducted so far [1, 4]. Abbiss and Laursen [5] describe six different pacing strategies such as (*i*) negative pacing (i.e. increase in speed over time), (*ii*) positive pacing (i.e. continuous slowing over time), (*iii*) all-out pacing (i.e. maximal speed possible), (*iv*) even pacing (i.e. same speed over time), (*v*) parabolic-shaped pacing (i.e. positive and negative pacing in different segments of the race) and (*vi*) variable pacing (i.e. pacing with multiple fluctuations). Pacing can be defined as the strategy by which effort is managed across an exercise bout in relation to a specific goal and in the knowledge of the likely demands of the task [6] or as the the process in which the total energy expenditure during exercise is regulated on a moment-to-moment basis in order to ensure that the exercise bout can be completed in a minimum time and without a catastrophic biological failure [7].

A review of studies examining the effect of sex and performance on pacing in endurance running and cycling concluded that athletes of higher performance level and women show a more even pacing than their counterparts with lower performance level and men, respectively [8]. In 10 and 15 km XC skiing races in World Cup, World Championships and Olympic events, slower men skiers were characterized by a relatively fast start, but no difference was found in women [9]. A study on a relatively small sample of finishers in the ‘Vasaloppet’ skiing race showed that women had a more even profile than men [4]. With regards to the effect of age on pacing, trivial differences among age groups have been previously observed in XC skiers [1, 4].

As a result of this limited available information on the variation of differences in pacing among performance groups by age, there is a gap in the existing knowledge with regards to the pacing strategies that master XC skiers should develop. Coaches may train a group of master XC skiers with different performance level and whether they should instruct them differently with regards to pacing remains still unanswered. It is also possible that coaches train XC skiers with similar race time but different age. In both cases, one should address the question of age×performance interaction on pacing. Thus, the aim of the present study was to examine the effects of age and performance on pacing in order to provide evidence-based suggestions for pacing in master XC skiers according to their performance level.

## Methods

To address the question of pacing in master XC skiers, we analysed the ‘Vasaloppet’, which is the oldest and longest cross-country ski race in the world and has the largest rates of participation [4]. The ‘Vasaloppet’ is 90 km and includes seven stations which together with the start and the finish define eight splits (www.vasaloppet.se/). Table 1 presents detailed information about stations and splits, e.g. distances and elevations.

**Table 1:** Stations, splits, their distance and elevation

	Distance (km)	Split (km)	Elevation (m)	Change in elevation (m)
Start	0	–	350	–
Station 1	11	11	480	130
Station 2	24	13	425	−55
Station 3	35	11	420	−5
Station 4	47	12	430	10
Station 5	62	15	230	−200
Station 6	71	9	250	20
Station 7	81	10	205	−45
End	90	9	165	−40

Two consecutive stations define a split, e.g. station 1 and station 2 define split 2. Change in elevation refers to a specific split and is calculated as the difference between the elevation of two successive stations. For instance the change in elevation during split 6 is the difference between station 5 and station 6, i.e. 250–230 = 20 m

We focused on the race versions from 2012 to 2017, as there were available age group of finishers and split times only for these calendar years. A total of 79,722 finishers (women, *n* = 9847; men, *n* = 69,875) were classified into age groups 19–20, 21–34, 35–39, 40–44, 45–49, 50–54, 55–59, 60–64, 65–69, 70–74, 75–79 and 80–84 year old, and performance groups based on their race time 3-4 h, 4-5 h, ..., 12-13 h. The pacing strategy was defined as the dependent variable, whereas sex, performance group and age group were the independent variables. Three pace parameters for each finisher were calculated [10]: a) positive pace range in the fastest split as 100 × (speed, km/h, in the fastest split - mean race speed, km/h)/mean race speed, km/h, e.g. + 22.0%, b) negative pace range in the slowest split as 100 × (speed, km/h, in the slowest split - mean race speed, km/h)/mean race speed, km/h, e.g. -16.2%, and c) total pace range as the absolute difference between positive and negative range, e.g. + 22.0% - (− 16.2%) = 38.2%.

All data are presented as means±standard deviations. Statistical analyses were carried out using GraphPad Prism v. 7.0 (GraphPad Software, San Diego, USA) and IBM SPSS v.23.0 (SPSS, Chicago, USA). Performance group×age group association was examined using chi-square (*χ*^*2*^) and the magnitude of the associations was tested by Cramer’s phi (*φ*). A two-way analysis of variance (ANOVA) examined the effects sex, performance group and age group on total, positive and negative pace range. Subsequent comparisons among groups were carried out using post-hoc Bonferroni test. The magnitude of the differences among groups was examined using effect size eta square (*η*^*2*^) and was evaluated as following: small (0.010 < *η*^*2*^ ≤ 0.059), moderate (0.059 < *η*^*2*^ ≤ 0.138) and large (*η*^*2*^ > 0.138) [11]. The acceptable type I error was set at *p* < 0.05.

## Results

Most women were in the 8-9 h performance group, whereas most men in the 6-7 h performance group (Table 2). A performance group×age group association was observed in women (χ^2^ = 340.4, *p* < 0.001, φ = 0.186), e.g. the 4-5 h performance group was 5.8% in the 21–34 age group and 2.0% in the 50–54 years age group. In addition, a performance group×age group association was shown in men, too (χ^2^ = 340.4, *p* < 0.001, φ = 0.186), e.g. the 4-5 h performance group was 10.2% in the 21–34 years age group and 2.8% in the 50–54 years age group.

**Table 2:** Finishers by sex, age and performance group

	Age group												
Performance group	19–20	21–34	35–39	40–44	45–49	50–54	55–59	60–64	65–69	70–74	75–79	80–84-	Total
*Women*													
3-4 h	–	–	–	–	–	–	–	–	–	–	–	–	–
4-5 h	8	227	47	59	47	20	2	2	0	0	–	–	412
5-6 h	27	352	118	172	100	64	20	8	2	0	–	–	863
6-7 h	24	489	155	241	209	137	65	12	6	0	–	–	1338
7-8 h	26	658	193	288	243	199	85	20	15	1	–	–	1728
8-9 h	31	690	217	312	294	226	98	40	18	7	–	–	1933
9-10 h	30	651	201	232	238	184	74	59	21	2	–	–	1692
10-11 h	25	647	180	182	196	169	84	36	8	4	–	–	1531
11-12 h	5	99	28	29	27	24	12	8	5	2	–	–	239
12-13 h	1	92	11	5	1	1	0	0	0	0	–	–	111
Total	177	3905	1150	1520	1355	1024	440	185	75	16	–	–	9847
*Men*													
3-4 h	0	105	26	6	3	0	0	0	0	0	0	0	140
4-5 h	29	1803	648	767	487	237	86	24	2	0	0	0	4083
5-6 h	97	2948	1754	2466	2126	1420	661	284	110	18	0	0	11,884
6-7 h	92	2786	1725	2339	2193	1639	979	496	275	84	7	0	12,615
7-8 h	88	2739	1539	2043	1935	1549	1016	624	392	144	37	2	12,108
8-9 h	82	2487	1294	1639	1605	1293	935	669	387	205	34	8	10,638
9-10 h	101	2134	1055	1286	1238	1043	699	568	345	194	44	14	8721
10-11 h	69	1521	668	738	722	668	417	352	190	123	35	9	5512
11-12 h	40	1062	438	484	443	419	273	215	134	75	30	6	3619
12-13 h	1	141	61	69	75	66	36	47	27	23	7	2	555
Total	599	17,726	9208	11,837	10,827	8334	5102	3279	1862	866	194	41	69,875

**Fig. 1:**
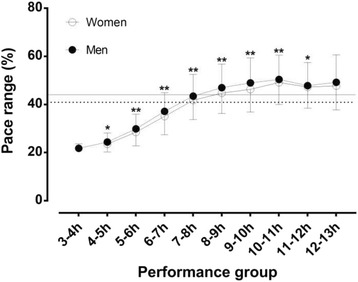
Total pace range by sex and performance group. Solid and dashed horizontal lines represent mean scores of women and men, respectively. There is no 3-4 h performance in women.*p < 0.05; **p < 0.001

A trivial main effect of sex on total pace range was observed (*p* < 0.001, η^2^ = 0.002), where women (44.1 ± 10.2%) had larger total pace range than men (40.9 ± 11.8%) (Fig. 1). A large main effect of performance group on total pace range was shown (*p* < 0.001, η^2^ = 0.160), where the smallest total pace range was 21.8 ± 1.9% (3-4 h group) and the largest 50.1 ± 9.9% (10-11 h group). A trivial sex×performance group interaction on total pace range was found (*p* < 0.001, η^2^ = 0.001) with the largest sex difference in pacing shown in the 9-10 h group.

**Fig. 2:**
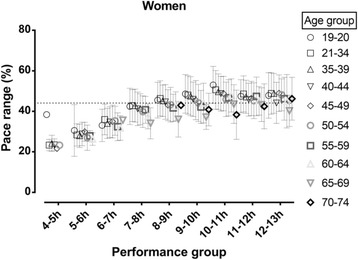
Total pace range by age and performance group in women. Dashed horizontal line represents mean score

**Fig. 3:**
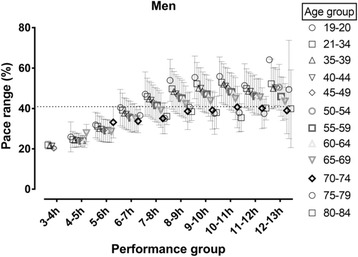
Total pace range by age and performance group in men. Dashed horizontal line represents mean score

In women, a trivial main effect of age on total pace range was observed (*p* < 0.001, η^2^ = 0.005) with smaller total range in the older age groups (Fig. 2). A small main effect of performance group on total pace range was shown (*p* < 0.001, η^2^ = 0.048) with the smallest total pace range in the fastest performance group. A trivial age group×performance group interaction on total pace range was found (*p* < 0.001, η^2^ = 0.008) with smaller differences among age groups in the faster performance group. In men, there was small main effect of age (*p* < 0.001, η^2^ = 0.011), moderate main effect of race time (*p* < 0.001, η^2^ = 0.065) and trivial age group×performance group interaction on total pace range (*p* < 0.001, η^2^ = 0.006), respectively, with similar trends as described for women (Fig. 3).

A trivial main effect of sex on positive pace range was observed (*p* < 0.001, η^2^ = 0.006), where men (17.88 ± 5.06%) had larger positive pace range than women (17.86 ± 4.47%) (Table 3). A moderate main effect of performance group on positive pace range was shown (*p* < 0.001, η^2^ = 0.091), where the smallest positive pace range was 12.1 ± 1.6% (3-4 h group) and the largest 21.2 ± 5.6% (10-11 h group). A trivial sex×performance group interaction on positive pace range was found (*p* < 0.001, η^2^ = 0.002) with the largest sex difference in positive pace range shown in 8-9 h group. Νο main effect of sex on negative pace range was observed (*p* = 0.452, η^2^ < 0.001) with women and men having − 26.2 ± 7.4% and − 23.0 ± 8.4%, respectively (Table 4). A moderate main effect of performance group on negative pace range was shown (*p* < 0.001, η^2^ = 0.124), where the smallest negative pace range was − 9.7 ± 1.4% (3-4 h group) and the largest − 28.3 ± 7.7% (> 12 h group). A trivial sex×performance group interaction on negative pace range was found (*p* < 0.001, η^2^ < 0.001) with the largest sex difference in negative pace range shown in 5-6 h group.

**Table 3:** Positive pace range (%) by sex, age and performance group

	Age group																							
	19–20		21–34		35–39		40–44		45–49		50–54		55–59		60–64		65–69		70–74		75–79		80–84	
	M	SD	M	SD	M	SD	M	SD	M	SD	M	SD	M	SD	M	SD	M	SD	M	SD	M	SD	M	SD
*Women*																								
3-4 h																								
4-5 h	16.0		12.4	1.8	12.7	2.2	12.3	0.9	7.2		14.1													
5-6 h	13.3	4.7	13.3	2.1	12.8	1.7	13.3	1.5	13.5	1.5	12.4	2.5	13.9	1.1	12.6	0.2								
6-7 h	14.3	2.7	15.0	2.4	14.5	2.4	14.4	2.5	14.5	2.7	14.6	2.4	13.6	2.0	13.9	3.2	15.3	1.9						
7-8 h	17.4	3.3	17.2	3.2	16.9	3.2	16.1	3.2	16.4	3.3	16.0	3.0	15.6	2.7	14.4	2.2	12.8	2.4						
8-9 h	19.7	4.6	18.5	3.9	17.9	3.7	17.5	3.4	17.0	3.8	16.7	3.4	15.8	2.9	16.1	2.8	16.0	3.3	13.6					
9-10 h	20.2	4.3	19.5	4.6	19.1	4.2	18.5	4.3	18.5	4.6	17.8	4.2	16.9	4.0	17.2	4.5	15.5	2.6	15.0	2.5				
10-11 h	23.1	5.6	20.8	5.7	20.3	4.7	20.1	4.8	19.5	4.0	18.2	4.1	18.5	4.2	18.6	4.3	18.1	3.6	15.7	6.1				
11-12 h	18.9	3.4	19.5	4.7	19.1	4.1	18.2	3.7	18.3	4.0	18.1	4.8	19.5	4.6	18.5	3.6	17.6	4.9	19.1	4.1				
12-13 h	17.7	2.3	20.3	5.0	18.6	4.7	18.1	3.9	20.2	4.4	18.8	3.6	18.4	4.8	18.8	6.8	18.7	4.0	15.5	5.5				
*Men*																								
3-4 h			12.2	1.7	11.6	1.3	11.4	1.0	10.6	1.3														
4-5 h	13.4	3.4	13.1	2.1	12.9	1.7	12.8	1.7	12.8	1.7	12.4	1.3	12.5	1.3	12.2	1.1	13.7	0.4						
5-6 h	15.0	2.9	14.3	2.5	14.0	2.3	13.8	2.3	13.6	2.2	13.4	2.2	13.4	2.0	13.3	2.0	13.0	1.9	14.1	2.9				
6-7 h	17.6	4.1	16.8	3.5	16.5	3.3	16.2	3.2	16.1	3.1	15.6	3.2	15.5	3.1	15.3	3.1	15.2	3.1	14.8	2.3	13.7	1.3		
7-8 h	20.1	4.5	19.5	4.2	19.3	4.0	19.1	4.1	18.7	4.1	18.3	4.1	18.1	3.9	17.5	4.1	17.5	4.0	15.5	3.7	16.3	3.9	18.0	2.6
8-9 h	23.6	5.1	20.9	4.9	20.6	4.8	20.2	4.7	20.1	4.9	19.4	4.7	19.3	4.8	19.3	5.0	18.9	4.5	17.7	4.5	19.5	4.8	18.9	4.5
9-10 h	24.1	6.3	22.2	5.4	21.3	5.3	21.2	5.0	20.7	5.2	20.3	5.1	20.1	5.0	19.5	5.2	19.4	5.1	17.9	5.2	19.0	5.8	18.1	5.8
10-11 h	24.9	6.5	22.8	6.1	22.1	5.9	21.6	5.1	21.5	5.6	20.6	5.2	20.5	4.8	20.1	4.9	19.5	5.7	18.3	4.8	18.6	6.5	17.7	5.9
11-12 h	21.1	5.0	20.6	5.5	20.2	5.3	20.3	4.7	19.5	4.7	19.4	5.2	19.4	4.6	19.3	4.7	19.2	5.5	17.3	4.4	17.3	4.2	19.6	4.1
12-13 h	28.6		21.7	6.3	21.1	4.3	21.0	5.2	21.3	4.9	21.6	6.2	20.3	4.1	18.6	4.4	19.0	4.9	19.5	6.5	22.1	10.3	20.7	5.2

**Table 4:** Negative pace range (%) by sex, age and performance group

	Age group																							
	19–20		21–34		35–39		40–44		45–49		50–54		55–59		60–64		65–69		70–74		75–79		80–84	
	M	SD	M	SD	M	SD	M	SD	M	SD	M	SD	M	SD	M	SD	M	SD	M	SD	M	SD	M	SD
*Women*																								
3-4 h																								
4-5 h	−22.4		−10.9	2.1	−11.6	2.4	−11.2	2.4	−14.4		−9.2													
5-6 h	−17.2	8.4	−15.1	4.3	−15.1	4.6	−15.5	4.8	−16.4	4.1	−14.6	4.5	−13.9	3.7	−13.7	3.4								
6-7 h	−18.7	6.4	−20.9	6.3	−19.5	6.0	−20.3	6.2	−20.4	6.4	−20.7	5.8	−18.8	5.8	−17.9	3.9	−20.4	0.8						
7-8 h	−25.0	7.1	−25.6	6.3	−25.9	6.5	−25.1	6.5	−24.7	6.0	−23.8	6.5	− 25.2	5.2	−23.2	5.0	−21.3	5.8						
8-9 h	−25.9	6.8	−27.9	6.9	−27.3	6.3	−27.2	6.9	−26.0	6.0	−26.6	6.8	−25.9	5.4	−26.4	5.9	−20.1	6.5	−29.3					
9-10 h	−28.4	7.9	−28.6	7.0	−28.4	7.1	−27.5	6.3	−27.0	7.2	−26.3	6.7	−25.2	6.6	−26.9	7.1	−21.7	5.9	−25.9	5.7				
10-11 h	−30.0	5.6	−29.8	6.4	− 29.4	6.1	−28.7	6.0	−29.3	6.6	−27.8	6.7	−28.2	6.4	−29.3	6.0	−25.4	7.7	−22.7	6.0				
11-12 h	−28.5	6.5	−29.1	6.1	−28.1	6.0	−27.6	6.3	−28.1	6.3	−27.1	5.9	−27.9	6.4	−26.7	6.2	−25.6	5.1	−23.3	7.4				
12-13 h	−30.1	9.7	−28.6	7.4	−30.3	6.6	−26.0	7.2	−28.7	7.4	−29.3	6.0	−27.8	6.6	−26.1	8.6	−21.5	5.5	−30.7	5.0				
*Men*																								
3-4 h			−9.6	1.4	−10.0	1.1	−10.0	1.4	−9.7	1.3														
4-5 h	−12.5	4.5	−11.5	2.8	−11.4	2.6	−11.5	2.4	−11.5	2.4	−11.4	2.3	− 11.4	1.8	− 11.3	2.3	−14.2	3.9						
5-6 h	−16.8	4.7	−16.9	5.0	−16.1	4.7	−15.9	4.7	−15.5	4.4	−15.5	4.3	−15.6	4.3	−15.4	4.3	− 15.7	4.2	−19.0	5.6				
6-7 h	−22.9	6.8	− 22.4	6.6	−21.2	6.1	− 21.0	6.1	−20.5	5.8	−20.3	5.9	−19.8	5.4	− 20.0	5.6	− 19.7	4.5	−18.9	4.9	−22.8	4.4		
7-8 h	−26.9	8.4	− 26.4	7.3	−25.4	7.0	−25.0	7.1	−24.4	6.8	−23.8	6.6	− 23.4	6.6	−22.8	6.6	−21.7	6.5	−19.6	5.9	−18.8	4.9	− 18.1	6.7
8-9 h	−30.3	8.2	−28.8	7.8	−27.8	7.8	−27.1	7.6	−26.5	7.2	− 26.3	7.2	−25.8	6.7	−24.8	7.0	−23.5	6.9	−21.0	6.9	− 21.1	6.7	−19.6	5.1
9-10 h	−31.3	6.9	−30.1	7.7	−28.7	7.7	− 28.8	7.7	−27.7	7.4	− 27.3	7.3	− 27.0	7.0	−25.7	6.8	−24.5	7.5	−21.3	6.9	−18.5	6.7	−19.9	4.7
10-11 h	−31.0	6.9	−30.2	6.9	− 30.0	6.9	−29.5	6.8	−28.9	6.9	− 28.0	7.0	−27.7	6.9	− 27.1	6.7	−25.4	7.8	−22.4	7.6	−19.2	6.0	−17.8	4.3
11-12 h	−30.3	7.6	−29.3	6.7	−28.4	6.7	− 28.2	6.6	−27.5	6.9	−27.3	6.9	−26.6	6.4	−26.5	6.6	−26.4	6.4	−22.8	7.9	−20.2	5.7	−20.5	8.6
12-13 h	−35.6		−30.5	7.9	−28.9	8.0	−29.3	6.8	−29.1	6.5	−28.9	6.9	−25.6	7.1	−26.1	8.3	−24.6	6.2	−19.5	8.0	−27.3	15.5	−19.2	14.1

## Discussion

The main findings of the present study were that (*i*) women had larger total pace range than men, (*ii*) the smallest total pace range was in the 3-4 h group and the largest in the 10-11 h group, (*iii*) the smallest total pace range was in the older age groups, and (*iv*) smaller differences among age groups were found in the faster performance groups.

The more even pacing observed in the older age groups was partially in agreement with previous studies on running. In 100-km ultra-marathon running, no differences in pacing among age groups was shown [12]. On the other hand, a more even pacing was found in older runners in the ‘New York City Marathon’ [13]. The smaller differences among age groups observed in the faster performance group was a novel finding as no previous study had ever examined the age×performance interaction on pacing in XC skiing.

We might assume that master XC skiers would exhibit different performance characteristics than their younger counterparts since aerobic capacity, which is a main determinant of performance in XC skiing [14], declines with aging [15]. A study that modelled changes in the criterion measure of aerobic capacity, i.e. maximal oxygen uptake (VO_2max_), identified age, fat-free mass and exercise training status as predictors of VO_2max_ [16]. Nevertheless, the decline of aerobic capacity with aging might be attenuated when large training volume is maintained in the master athletes [15, 17] and healthy adults [18]. Also, exercise might attenuate the decline of motor and cognitive abilities with aging [19]. Furthermore, it has been shown that the decline in VO_2max_ was proportional to the decline in training volume in endurance trained men [20]. Thus, the abovementioned long-term exercise-induced adaptations of aerobic capacity might consist in the physiological mechanism which attenuated the decline of performance with aging and explained why both younger and older fast XC skiers adopted similar pacing strategies.

In addition, the total, positive and negative pace range were smaller in the performance groups with fastest race time indicating that the faster performance groups adopted a more even pacing. This observation was in agreement with previous research [8] indicating that XC skiing presents similar trends of the relationship between performance and pacing as those observed in other endurance sports.

An unexpected result was that men showed a more even pacing than women, since research on endurance sports such as the ‘Chicago Marathon’ [21] and 100-km running [22] had previously suggested women as better pacers. This disagreement in the sex difference in pacing among endurance sports might be due to unique characteristics of XC skiing, where human body interacts with sophisticated equipment and there are increased demands in upper body muscle power compared to other running or cycling [23, 24]. In addition, the more even pacing in men observed in the present study was in contrast with a previous study on a small sample of finishers in the ‘Vasaloppet’, where women showed a more even pacing profile than men with the same finish time, start group, age, and race experience, and men were faster in the first half and women were faster in the second half of the race [4].

The results of the present study are limited by the unique characteristics of the ‘Vasaloppet’ in terms of race distance and change of elevation; therefore, they should be interpreted with caution when comparing with other XC races. Also, a unique characteristic of this study was the use of pace range to study pacing, which was a recently developed methodological approach [10]. Although this approach provided a reasonable estimate of pacing, as it identified accurately the slowest and fastest splits and the deviation (%) of their speed from the average race speed, the findings should not be compared to studies using other methodological approaches (e.g. coefficient of variation [25], % change of speed between consecutive splits [13]). Moreover, the findings were based on comparison among different sex, age and performance groups and, consequently, did not establish a causal relationship of sex, age and performance with pacing.

Nonetheless, strength of the study was the inclusion of all editions of the ‘Vasaloppet’ (2012–2017) for which all split times and finishers’ age were available resulting in one of the largest sample of XC skiers ever studied. The large number of finishers allowed drawing safe conclusions about differences in pacing by sex, age and performance group. It should be highlighted that the large sample size should be accounted for the statistically significant findings (e.g. at *p* < 0.001) in cases of trivial magnitude of differences; thus, both statistics (i.e. *p* value and effect size) should be considered in the interpretation of the findings. Furthermore, we highlighted unique pacing patterns in XC skiing which differ from other endurance sports such as the sex effect on pacing. Men XC skiers have a more even pacing than women which is in contrast with the sex trends in pacing in other endurance sports (e.g. running) that suggest women as better pacers. In XC skiing, the sex difference in pacing seems performance-dependent with men showing more even pacing than women in all performance groups, except the slowest.

Considering the large number of master XC skiers, the findings of the present study would be of practical importance for coaches and fitness trainers in this sport in order to adapt the training and competition practice such as pacing, which was previously established in younger XC skiers, in the specific demands of the master XC skiers. Fast master XC skiers should be advised adopting a similar pacing strategy as their younger counterparts. Older XC skiers should be expected to show a more even pacing than their younger counterparts. A trend of a relatively even pacing in both slow and fast older XC skiers compared to their younger counterparts should be taken into account by coaches and fitness trainers during the training practice and the preparation for a race such as ‘Vasaloppet’. By definition, pacing refers to the management of effort [6] or energy expenditure [7] during an exercise; thus, the variation of pacing by age might provide practical information for master athletes to optimize performance. The role of pacing is even more pronounced in the case of XC skiing, which is an ultra-endurance sport (i.e. race time longer than 6 h for most master finishers).

## Conclusions

In summary, the faster the race time or the older the XC skiers, the lower the total pace range (i.e. a relatively more even pacing was observed), which confirmed these trends previously shown in other endurance sports such as marathon running. What is novel is that based on the findings of the present study we identified an effect of age on pacing that depended on the performance level of XC skiers in the ‘Vasaloppet’, i.e. the better the performance, the smaller the differences in pacing among age groups. These findings suggest that endurance exercise may result in similar performance-related characteristics, e.g. pacing, in fast master XC skiers as in their younger counterparts.

## References

[1] Nikolaidis PT, Knechtle B (2017). Pacing profiles in age group cross-country skiers in the Vasaloppet 2012–2016. Chin J Physiol.

[2] Nikolaidis PT, Knechtle B (2018). The age-related performance decline in marathon cross-country skiing – the Engadin ski marathon. J Sports Sci.

[3] Knechtle B, Nikolaidis PT (2018). The age of peak marathon performance in cross-country skiing – the ‘Engadin ski marathon’. J Strength Cond Res..

[4] Carlsson M, Assarsson H, Carlsson T (2016). The influence of sex, age, and race experience on pacing profiles during the 90 km Vasaloppet ski race. Open Access J Sports Med..

[5] Abbiss CR, Laursen PB (2008). Describing and understanding pacing strategies during athletic competition. Sports Med.

[6] Edwards A, Polman R (2012). Pacing in sport and exercise: a psychophysiological perspective.

[7] Baron B, Moullan F, Deruelle F, Noakes TD (2011). The role of emotions on pacing strategies and performance in middle and long duration sport events. Br J Sports Med.

[8] Thiel C, Foster C, Banzer W, de Koning J (2012). Pacing in Olympic track races: competitive tactics versus best performance strategy. J Sports Sci.

[9] Losnegard T, Kjeldsen K, Skattebo Ø (2016). An analysis of the pacing strategies adopted by elite cross-country skiers. J Strength Cond Res.

[10] Breen D, Norris M, Healy R, Anderson R. Marathon pace control in masters athletes. Int J Sports Physiol Perform. 2017; doi.org/10.1123/ijspp.2016-0730 10.1123/ijspp.2016-0730 28714744

[11] Cohen J (1988). Statistical power analysis for the behavioral sciences.

[12] Rust CA, Rosemann T, Zingg MA, Knechtle B (2015). Do non-elite older runners slow down more than younger runners in a 100 km ultra-marathon?. BMC Sports Sci Med Rehab.

[13] Nikolaidis PT, Knechtle B (2017). Effect of age and performance on pacing of marathon runners. Open Access J Sports Med.

[14] Tonnessen E, Haugen TA, Hem E, Leirstein S, Seiler S (2015). Maximal aerobic capacity in the winter-Olympics endurance disciplines: Olympic-medal benchmarks for the time period 1990-2013. Int J Sports Physiol Perform.

[15] Rogers MA, Hagberg JM, Martin WH, Ehsani AA, Holloszy JO (1990). Decline in VO2max with aging in master athletes and sedentary men. J Appl Physiol.

[16] Rosen MJ, Sorkin JD, Goldberg AP, Hagberg JM, Katzel LI (1998). Predictors of age-associated decline in maximal aerobic capacity: a comparison of four statistical models. J Appl Physiol.

[17] Kusy K, Zielinski J (2014). Aerobic capacity in speed-power athletes aged 20-90 years vs endurance runners and untrained participants. Scand J Med Sci Sports.

[18] Padilla J, Eguía Lis MC, Licea J, Taylor AW (1998). Maximal aerobic capacity and sports activity in Mexicans from 13 to 56. Arch Inst Cardiol Mex.

[19] Levin O, Netz Y, Ziv G (2017). The beneficial effects of different types of exercise interventions on motor and cognitive functions in older age: a systematic review. Eur Rev Aging Phys Act.

[20] Pimentel AE, Gentile CL, Tanaka H, Seals DR, Gates PE (2003). Greater rate of decline in maximal aerobic capacity with age in endurance-trained than in sedentary men. J Appl Physiol.

[21] Trubee NW, Vanderburgh PM, Diestelkamp WS, Jackson KJ (2014). Effects of heat stress and sex on pacing in marathon runners. J Strength Cond Res..

[22] Renfree A, Crivoi do Carmo E, Martin L (2016). The influence of performance level, age and gender on pacing strategy during a 100-km ultramarathon. Eur J Sport Sci.

[23] Bjorklund G, Alricsson M, Svantesson U (2017). Using bilateral functional and anthropometric tests to define symmetry in cross-country skiers. J Hum Kinet.

[24] Østerås S, Welde B, Danielsen J, Van Den Tillaar R, Ettema G, Sandbakk O (2016). Contribution of upper-body strength, body composition, and maximal oxygen uptake to predict double poling power and overall performance in female cross-country skiers. J Strength Cond Res..

[25] Santos-Lozano A, Collado PS, Foster C, Lucia A, Garatachea N (2014). Influence of sex and level on marathon pacing strategy. Insights from the new York City race. Int J Sports Med.

